# A qualitative feasibility study to inform a randomised controlled trial of fluid bolus therapy in septic shock

**DOI:** 10.1136/archdischild-2016-312515

**Published:** 2017-08-28

**Authors:** Caitlin B O’Hara, Ruth R Canter, Paul R Mouncey, Anjali Carter, Nicola Jones, Simon Nadel, Mark J Peters, Mark D Lyttle, David A Harrison, Kathryn M Rowan, David Inwald, Kerry Woolfall

**Affiliations:** 1 Institute of Psychology, Health and Society, University of Liverpool, Liverpool, UK; 2 Clinical Trials Unit, Intensive Care National Audit and Research Centre (ICNARC), London, UK; 3 Patient and Public Involvement Partner, Watford, UK; 4 Paediatric Intensive Care Unit, St Mary’s Hospital, Imperial College Healthcare NHS Trust, London, UK; 5 Institute of Child Health, University College London, UK and Great Ormond Street Hospital NHS Foundation Trust, London, UK; 6 Faculty of Health and Applied Sciences, University of the West of England, Bristol, UK; 7 Emergency Department, Bristol Royal Hospital for Children, Upper Maudlin Street, Bristol, UK

**Keywords:** qualitative research, ethics, intensive care, sepsis

## Abstract

**Objective:**

The Fluids in Shock (FiSh) Trial proposes to evaluate whether restrictive fluid bolus therapy (10 mL/kg) is more beneficial than current recommended practice (20 mL/kg) in the resuscitation of children with septic shock in the UK. This qualitative feasibility study aimed to explore acceptability of the FiSh Trial, including research without prior consent (RWPC), potential barriers to recruitment and participant information for a pilot trial.

**Design:**

Qualitative interview study involving parents of children who had presented to a UK emergency department or been admitted to a paediatric intensive care unit with severe infection in the previous 3 years.

**Participants:**

Twenty-one parents (seven bereaved) were interviewed 16 (median) months since their child’s hospital admission (range: 1–41).

**Results:**

All parents said they would have provided consent for the use of their child’s data in the FiSh Trial. The majority were unfamiliar with RWPC, yet supported its use. Parents were initially concerned about the change from currently recommended treatment, yet were reassured by explanations of the current evidence base, fluid bolus therapy and monitoring procedures. Parents made recommendations about the timing of the research discussion and content of participant information. Bereaved parents stated that recruiters should not discuss research immediately after a child’s death, but supported a personalised postal ‘opt-out’ approach to consent.

**Conclusions:**

Findings show that parents whose child has experienced severe infection supported the proposed FiSh Trial, including the use of RWPC. Parents’ views informed the development of the pilot trial protocol and site staff training.

**Trial registration number:**

ISRCTN15244462—results.

**What is already known on this topic?**

- Research is needed to determine whether restrictive fluid bolus therapy is more beneficial than current recommended practice in the resuscitation of children with presumed septic shock.
- Paediatric emergency and critical care trials encounter practical and ethical difficulties, as there is no time to seek informed consent in an emergency situation.
- Feasibility work incorporating patient perspectives can help test key parameters and ensure the trial is appropriate to the needs of patients and their families.

**What this study adds?**

- Our findings support the proposed Fluids in Shock (FiSh) pilot trial and demonstrate the value of using qualitative methods to involve parents in the design of trials.
- Tailored verbal information can help address parents’ priorities, concerns and misconceptions about FiSh.
- This study provides new insight into what should happen if a child dies after being entered into a trial without prior informed consent.

## Introduction

Qualitative research to incorporate patient perspectives in the design of a clinical trial can help ensure the trial is acceptable and appropriate to the needs of patients and their families.^1^ This can increase the impact of the work, thus benefiting future trials and patient-centred healthcare.^2 3^

Paediatric trials in emergency and critical care settings can be challenged by the difficulties of seeking prospective informed consent for trial participation^4 5^ as time-critical interventions cannot be delayed.^6^ So that vital research can proceed, clinical trials legislation has been amended^7–11^ to enable children to be entered into a trial without prior informed consent.^4 12^ This is research without prior consent (RWPC), also called deferred consent, and it entails seeking consent postintervention for the use of data already collected and continued study participation.^10 13^ Although RWPC has been subject to debate,^14–16^ recent studies have indicated parental support for this approach.^1 12 17–20^ However, further research is needed to explore parent and practitioner views on using RWPC in trials relating to more complex situations, such as comparing usual clinical care with a change in care, or when a child dies after being entered into a trial.^1^

Rapid fluid replacement by bolus is integral to the management of children presenting to UK emergency departments (EDs) with septic shock. Current UK guidance, recommending fluid boluses of 20 mL/kg,^21^ is based on weak evidence.^22–24^ The Fluid Expansion as Supportive Therapy (FEAST) trial^25^ compared fluid bolus resuscitation of 20–40 mL/kg with no bolus maintenance fluid in over 3000 African children with severe infection and reported 30% higher mortality with fluid bolus resuscitation.^25^ This raised uncertainty and highlighted the lack of evidence for fluid bolus resuscitation for children in middle-income and high-income settings.

Accordingly, the Fluids in Shock (FiSh) randomised controlled trial (RCT) was designed to determine whether restrictive fluid bolus therapy (10 mL/kg) is more beneficial than current UK recommended practice (20 mL/kg). Prior to conducting the definitive RCT, the need for qualitative feasibility and clinical pilot work was identified to explore key challenges, including insufficient time to obtain informed consent and delivery of a fluid bolus protocol, which deviates from current recommended practice. This paper presents findings from the qualitative feasibility study, which aimed to review and explore with parents the acceptability of the FiSh Trial, approach to consent, potential barriers to recruitment and participant information materials for the pilot trial.

## Methods

### Study design

We conducted semistructured telephone interviews with bereaved and non-bereaved parents of children who had presented to a UK ED or been admitted to a paediatric intensive care unit (PICU) with severe infection in the previous 3 years. Severe infection was defined as any condition leading to treatment for sepsis or septic shock, for example, meningococcal septicaemia. Parents were excluded if they did not speak English. We used previous research^1 12 26^ to develop an interview topic guide (see online supplementary file 1 table 1) and draft Participant Information Sheet (PIS) for the pilot trial (see online supplementary file 2). The topic guide contained open-ended questions and prompts to help explore parents’ views on the acceptability of the FiSh Trial, including the pilot trial PIS and approach to consent. A separate section of questions was developed for bereaved parents.

10.1136/archdischild-2016-312515.supp1 Supplementary file 1 supplementary data

10.1136/archdischild-2016-312515.supp2 Supplementary file 2 supplementary data

### Recruitment and sampling procedure

**Figure 1:**
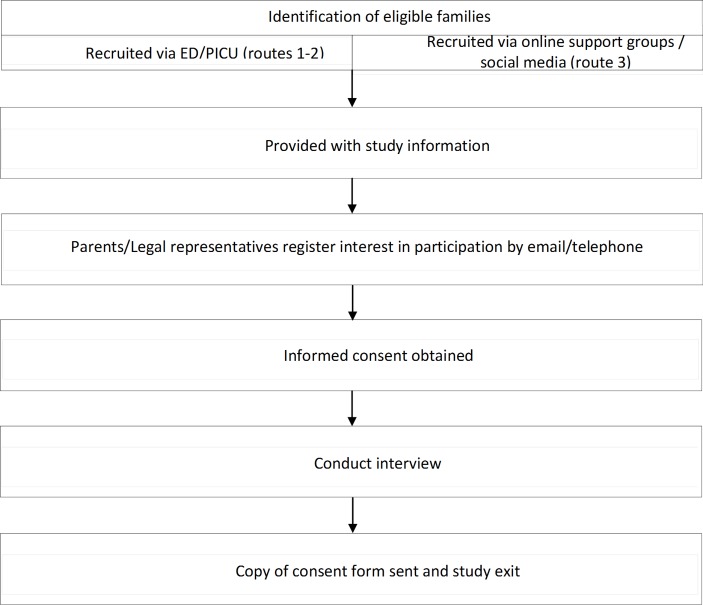
Fluids in Shock qualitative study flow chart. ED, emergency department; PICU, paediatric intensive care unit.

Based on previous studies,^1^ we anticipated recruiting 15–25 parents. We used three recruitment routes (figure 1).

#### Recruitment route 1: postal contact

Clinicians used hospital medical records to identify the 15 most recent parents (including up to five bereaved) of children who met the inclusion criteria. Those identified were sent a postal invitation, including a covering letter and qualitative study PIS that detailed how to register interest in taking part.

#### Recruitment route 2: advertising in PICU

A participant information poster and copies of the qualitative study PIS were placed in PICU family rooms in hospitals.

#### Recruitment route 3: advertising online including social media

We posted an advert on Twitter and Facebook and asked relevant charities and parent support groups to place the advert on their website and social media.

### Eligibility screening and interview conduct

CBO and KW responded to parents’ requests to participate in sequential order, confirmed eligibility and emailed them the pilot trial PIS. Audio-recorded verbal consent was sought before the interview began.

Digital audio recordings were transcribed verbatim, anonymised and checked for accuracy. Respondent validation was used to add unanticipated topics to the topic guide as interviewing and analysis progressed.^27^ Interviews aimed for data saturation, that is, the point where no new major themes are discovered in analysis.^28 29^ Screening stopped when data saturation^28 29^ was reached. All participants received a £30 shopping voucher and letter thanking them for their time.

### Analysis

CBO (a psychologist) led the analysis with assistance from KW (a sociologist). Analysis was broadly interpretive and iterative^30 31^ (see online supplementary file 3 table 2). Informed by the constant comparative approach, the aim was to provide accurate representation of parental views on trial design and acceptability.^32–35^ We used NVivo V.10 software to assist in the organisation and coding of data.

10.1136/archdischild-2016-312515.supp3 Supplementary file 3 supplementary data

## Results

### Sample

**Figure 2:**
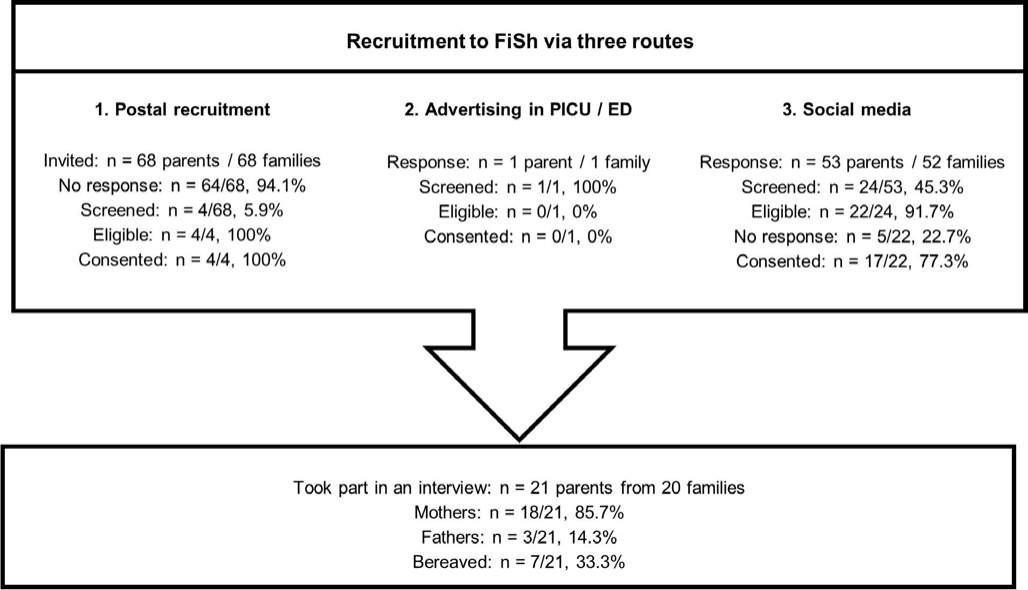
FiSh qualitative study parent recruitment process. For families identified using social media, responses were dealt with in the order they were received, screened, then interviewed where eligible. Data saturation was reached without the need to screen the remaining responses. ED, emergency department; FiSh, Fluids in Shock; PICU, paediatric intensive care unit.

Four UK hospitals took part in recruitment routes 1 and 2, and 11 charities/support groups in recruitment route 3. A total of 58 parents registered interest, of whom 29 were screened (figure 2); three were deemed ineligible and five did not confirm a date for interview. Data saturation^28 29^ was reached when 21 parents were interviewed (18 mothers (5 bereaved), 3 fathers (2 bereaved)).

Bereaved parents were interviewed on average 11 (median) months since admission (range: 3–31) and non-bereaved parents on average 16.5 months since admission (range: 1–41). The median hospital length of stay for their child was 1 day (range: 1–25) for bereaved parents and 14 days (range: 4–140) for non-bereaved parents. Eight parents had previously participated in a clinical trial. One mother had experience of RWPC. Interviews took between 30 and 55 min (see online supplementary file 4 table 3 for selected quotations from parents by theme).

10.1136/archdischild-2016-312515.supp4 Supplementary file 4 supplementary data

### RWPC is acceptable but with some initial concerns

A general definition of RWPC was first read to parents (box 1). Many described how they may be initially surprised to discover that their child had been entered into a trial without their prior consent. However, concerns subsided once reasons for using RWPC in emergency situations were considered. Parents went on to respond favourably to RWPC, describing it as a logical solution to enable research in challenging circumstances.

Box 1 **Description of research without prior consent read to participants during interview**

Due to the need to treat a patient in an emergency without delay, or because parents may not always be present when a child needs treatment, it is not always appropriate or possible to obtain consent before a child is entered into a trial. To enable research to be conducted in the emergency setting, many countries (including the UK) allow consent to be sought as soon as possible afterwards. This is for permission to use the data already collected and to continue in the trial. This is research without prior consent (sometimes called deferred consent). Research without prior consent is a relatively new approach to seeking consent in the UK.

### Support for FiSh Trial but with some concerns and misconceptions

Overall, parents spoke of their support for FiSh and its use of RWPC. Many viewed trial participation as a way to help other families and children in the future. Some parents thought that a recruitment discussion could provide a *“distraction”* (P9, mother, non-bereaved) and foster *“a sense of control in a situation where you feel completely out of control”* (P6, mother, non-bereaved).

However, support for the FiSh Trial appeared to be dependent on the intervention being successful and *“how well [their child] was”* (P4, mother, non-bereaved). Parents, including those who were bereaved, said they would question whether their child’s participation in the trial was “*the reason [their child] didn’t survive”* (P19, mother, bereaved), in that eventuality.

Some parents held specific concerns and misconceptions, which influenced their views on the acceptability of the FiSh Trial. Many were concerned that a change from current practice may jeopardise their child’s chances of survival. This was linked to the misconception that routinely used treatments are proven to be effective. Parents were also concerned that restrictive fluid bolus therapy would be insufficient as a liberal approach to fluid bolus resuscitation was viewed as being more likely to save a child’s life.

In response, CBO directed parents to sections of the pilot trial PIS and provided tailored explanations, such as the weak evidence base for current recommended practice, how fluid resuscitation is part of a larger treatment package and monitoring procedures. Such tailored explanations appeared to address parents’ initial concerns and misconceptions about the proposed FiSh Trial.

### Unclear or missing study information

All parents described the pilot trial PIS as being clear and concise. However, parents raised questions about aspects of FiSh (box 2), indicating that key information was unclear or missing. Importantly, this information was prioritised by parents, impacting on their understanding and views about the acceptability of the trial. Many said they would not ask questions or raise concerns with a FiSh recruiter.

Box 2 **Examples of questions raised by parents**

- Would the amount of fluid given be corrected if my child was not stabilising?
- Does the amount of fluid have a direct impact on outcome?
- What is a fluid bolus?
- What is the timing of fluid bolus administrations?
- Will I be able to find out which group my child was randomised to?
- Does the fluid treatment apply regardless of the child’s age?

### When to approach parents to discuss the FiSh Trial

Parents described how a FiSh Trial discussion should happen after the initial stress had subsided and their child’s condition had stabilised, preferably within 24–48 hours. Parents expressed the need for recruiters to gauge appropriate timing of this discussion in consultation with the clinical team.

We asked bereaved parents to consider a scenario in which their child had been entered into FiSh before death and a practitioner approached them after death to discuss the trial. They suggested that FiSh recruiters should be prepared to address concerns about whether trial participation was *“a reason as to why [death] happened”* (P21, mother, bereaved). They emphasised a need for sensitivity and time, particularly if their child had died very quickly, without warning. Parents described their anger in the initial stages of bereavement, which they believed would negatively impact on decision-making abilities and their response to a FiSh Trial discussion. Nevertheless, all bereaved parents agreed it would be acceptable to discuss FiSh at a later time, after they had left hospital. Bereaved parents valued medical research and described how they would have consented to the use of their child’s data as a way to help other children in the future.

We then sought bereaved parents’ views on the most appropriate way of contacting parents to discuss FiSh following death of a child (box 3). Several parents thought an appropriately timed face-to-face discussion with a nurse or consultant would be preferable to a telephone call or letter. The majority however supported contact via post at 4 weeks and then at 8 weeks after death as long as the ‘opt-out’ approach provided was emphasised in bold and the letter personalised, ideally by a known member of the clinical team. However, parents described grief as a *“very personal matter”* (P17, mother, bereaved), making it difficult to develop general recommendations on how best to approach parents in this situation.

Box 3 **Options for approaching bereaved parents to discuss the Fluids in Shock Trial after a child has died**

The researcher presented several options to consider:

- Face-to-face discussion with a nurse or doctor
- Telephone call by a nurse or doctor
- Personalised letter 4 weeks after randomisation, followed by a second letter 8 weeks after randomisation (ie, if no response is received after sending the initial letter); letters would explain how to opt out of the study and that there would be no need to respond if they wanted their child’s data to be used in the trial

## Discussion

This study provides insight into the acceptability of the FiSh Trial by exploring the views of parents with relevant experience. Consistent with the CONNECT  (CONseNt methods in paediatric Emergency and urgent Care Trials) study findings^1 12^ and associated guidance on RWPC,^36 37^ some parents were initially surprised about the concept of RWPC. However, initial concerns subsided when reasons why informed consent could not be sought were considered. As also shown in previous international research,^18 38^ parents questioned their ability to provide a rational, informed decision about research in an emergency situation^1 4 18 39^ and supported alternative approaches to prospective informed consent as a way to enable research in time-critical situations to improve treatments for critically ill children.^1 18 38 40^

Our findings highlight specific concerns and misunderstandings, which initially influenced parental views on acceptability of the FiSh Trial.^19^ Concerns included the proposed change from current clinical practice and its potential impact on a child’s recovery. Although the pilot PIS included a description of the weak evidence for current practice, many held a misconception that this was the proven optimal treatment. Moreover, several parents were unclear about the nature of fluid bolus therapy and only one understood that 20 mL/kg would be given to all children before being entered into FiSh. Nevertheless, all parents reported they would have provided consent for the use of their child’s data in FiSh, valuing the opportunity to advance medical research and viewing their child’s participation as a means to help similar families in the future.^1^ This is consistent with previous findings that emphasise the need for simple, non-medicalised information to improve parental understanding of trial information and inform their research decisions.^1 26 41^ Tailored explanations appeared to address parents’ priorities, concerns and misconceptions. These findings were used to develop the FiSh pilot trial site staff training, which emphasised the need for recruiters to provide opportunities for questions, as parents are unlikely to voice potential concerns.^19 42^

Consistent with previous studies that have explored approaches to consent in time-critical situations,^1 18 40 43 44^ the timing of the recruitment discussion was found likely to impact on parental responses to FiSh. Although many parents described how consultation with the clinical team would help recruiters gauge when to approach families to discuss the trial,^37^ their views on when and how this initial contact should happen differed, depending on whether or not their child survived. Parents of children who had survived severe infection expressed how they would wish to be approached in hospital, ideally within 24–48 hours of randomisation, once their child’s condition had stabilised.^36^ In contrast, bereaved parents emphasised that parents in this situation should not be approached immediately after their child’s death, as this may heighten feelings of grief and anger. It was this perceived burden of having a research discussion with vulnerable families that led to a waiver of consent (ie, no trial discussion) when a child died in FEAST.^18 40 45^ However, our findings do not support the model used in this study,^17^ as bereaved parents described how they would wish to discuss the use of their child’s information in FiSh, as long as the timing of this discussion was appropriate.^12 37^

Our findings provide new insight into what should happen if a child dies after being entered into a trial.^36^ Although some bereaved parents preferred a face-to-face research discussion, the majority responded favourably to being contacted via a letter, providing the opportunity to meet with a practitioner at a later date. Bereaved parents responded favourably to the inclusion of their child’s data in the trial unless the family notify the research team, provided this ‘opt-out’ approach is emphasised in bold text within the letter. Importantly, parents recommended that the letter should be sent from a practitioner known to the family with whom they had developed a close and trusting rapport.^36^ As septic shock is associated with an 8%–17% mortality rate,^23 46^ these findings are particularly important and will help design a trial that is appropriate to the needs of vulnerable families. The views of bereaved parents also highlight the need for practitioners to prepare to respond to parents who are concerned that trial participation may have resulted in harm.

### Strengths, limitations and future implications

While our sample size was relatively small, data saturation was reached^35^ and both bereaved and non-bereaved parents were included. As the majority of parents were recruited using social media, our sample may comprise parents with an interest in research and may not reflect the potential FiSh sample. Although all parents had experience of paediatric emergency and critical care, the majority had no previous experience of RWPC. Additional qualitative research will therefore be conducted within the FiSh pilot trial to further explore parental acceptability of the FiSh Trial, approach to consent and practitioner training needs.

Our findings add to the existing literature on RWPC in paediatric emergency and critical care settings. As such, they provide an important contribution and demonstrate the value of using qualitative methods in considering family-centred parameters needed to inform the design and conduct of challenging trials.^47 48^

Finally, children were not involved in this study. Research is required to explore children’s views on RWPC in emergency and critical care trials.

## Conclusions

Overall, findings suggest that parents whose child has experienced severe infection support the proposed FiSh Trial, including the use of RWPC. Parents’ views have informed the development of the pilot trial protocol and site initiation training.

10.1136/archdischild-2016-312515.supp5 Supplementary file 5 supplementary data
