# Supplementary material for: A qualitative feasibility study to inform a randomised controlled trial of fluid bolus therapy in septic shock
Source: Arch Dis Child. 2017 Aug 28;103(1):28–32. doi: 10.1136/archdischild-2016-312515 (PMC5754873; doi:10.1136/archdischild-2016-312515)
Supplement: Supplementary file 1 [file archdischild-2016-312515supp001.docx]

**Table 1.** Example interview topics and questions

| Interview topics | Example questions |
| --- | --- |
| Knowledge and experience | *Could you give me an outline of what happened to your child for them to need emergency treatment for severe infection?*    *Do you know if your child was given a fluid bolus?*  *Have you ever heard of a clinical trial before?*  *Have you ever been asked if your child could take part in a clinical trial?* |
| Participant information | *Looking at the information sheet, are there any parts of the study that you think parents may find difficult to understand?*  *Would you have any questions about the FiSh Trial?*  *Would you have any concerns about the FiSh Trial?* |
|  |  |
| Consent decision-making | *Are there sections of the FiSh information sheet which you would prioritise when making your decision about whether or not to consent?*  *How much time would you need to consider the information before making a decision about the FiSh Trial?* |
| Approach to consent | *[Following the description in Box 1]*  *Have you ever heard of research without prior consent (RWPC) before?*  *What do you think about the use of RWPC in the proposed FiSh Trial?*  *When do you think is the best time to approach a family to discuss the FiSh Trial?* |
|  |  |
| Participation | *Would you have given your permission for the use of your child’s data in the FiSh Trial?*  *[Prompt] Could you tell me a bit more about your reasons for this?* |
|  |  |
| For bereaved parents only | *What do you think about approaching parents for consent for their child’s data after they have passed away?*  *What advice would you give doctors and nurses on how to go about approaching bereaved parents for consent for the FiSh Trial?*  *How do you think this should be done? [Prompt: explore: face-to-face, telephone call, letter]*  *When do you think is the best time for doctors and nurses to approach bereaved parents for consent?* |
