# Supplementary material for: A qualitative feasibility study to inform a randomised controlled trial of fluid bolus therapy in septic shock
Source: Arch Dis Child. 2017 Aug 28;103(1):28–32. doi: 10.1136/archdischild-2016-312515 (PMC5754873; doi:10.1136/archdischild-2016-312515)
Supplement: Supplementary file 2 [file archdischild-2016-312515supp002.pdf]

Fluids in Shock (FiSh) Pilot Study

(FOR USE IN FEASIBILITY STUDY INTERVIEWS)

Insert NHS logo
<Trust address 1> <Trust address 2>
<Trust address 3> <postcode>
<telephone number>
Centre Number: <centre number>

Parent/Legal Representative Information Sheet

Contents

	Page
1) Why are we doing this study?	2
2) What do I need to know about the treatments used in this study?	2
3) How was it decided which fluid amount my child received?	2
4) Why am I being asked *after* my child has been given the treatment rather than before?	2
5) What will happen next?	2
6) Who is involved in this study?	3
7) What if there is a problem?	3
8) Who has reviewed the study?	3

How to contact us

If you have any questions please contact:

Research Nurse
Name: **name**
Telephone: **number**

Principle Investigator
Name: **name**
Telephone: **number**

We invite you to provide consent for your child/relative's information to be used in a research study

Children are now much more likely to survive any serious infection than ever before. This is a result of a whole package of treatments including antibiotic, infusions of fluid (saline solution) into a child's veins ("fluid bolus therapy") and support for breathing and heart function. We hope to improve survival further by refining one part of this package of treatment - by exploring *what is the best amount of fluid to give critically ill children with severe infection in the earliest stages of care*.

Findings from a recent large African trial suggest that less fluid bolus therapy might be a better in treating children with severe infection (septic shock) than the amount currently recommended in the UK. These UK recommendations come from expert opinion and have not been proven in a clinical trial. Therefore, further research is needed to find out which amount is best at treating septic shock in children.

We are doing a study (called FiSh – Fluids in shock) to find out whether giving less fluid (10 millilitres (ml) per kilogramme (kg)) to children with severe infection is better than giving a higher (20 ml per kg) amount of fluid. This is a small study which is being done to inform a bigger clinical trial.

Before you decide if you want to give your permission for your child/relative's information to be used in this study, it is important for you to understand why the research is being done and what it will involve. Please take the time to read the following information carefully. Discuss it with friends and relatives if you wish.

You are free to decide whether or not you wish for your child/relative's information to be used. Your decision will not affect the care your child/relative will receive.

Please ask the nurse or doctor who has spoken to you about the FiSh Pilot Study if there is anything that is not clear or if you would like more information.

Important things that you need to know

- Your child/relative was showing signs of severe infection (called septic shock). It was important to treat your child as quickly as possible.
- Across the UK, children with septic shock are treated by injecting fluid into a vein. This is called fluid bolus therapy.
- In this study we want to find out whether giving children a fluid bolus of 10 ml per kg (about 2 medicine spoons full) is better than the usual amount given (20 ml per kg).
- As this was a medical emergency, your child/relative has already been given either 10 ml or 20 ml per kg of fluid as there was no time to delay their treatment.

1) Why are we doing this study?

Septic shock is a life-threatening condition that happens when a child's blood pressure drops to a dangerously low level due to bacterial infection**.** Symptoms can include a high temperature, raised pulse rate, quick breathing and confusion. Previously healthy children may develop septic shock, sometimes very quickly, due to bacterial infection. Some children have a higher risk than others, including those with a lowered immune system (e.g. due to leukaemia or chemotherapy) and those with various long term medical problems.

We want to find out whether children with symptoms of septic shock should be treated with less fluid (10 millilitres (ml) per kilogram (kg)) than is currently recommended (20 ml per kg). This kind of study is the best way of finding out.

This hospital is one of 12 that are taking part in this study across the country. The study will involve approximately 108 children and young people. This study is being done to inform a larger clinical trial which we plan to run in hospitals across the UK.

2) What do I need to know about the treatments used this study and possible side effects?

Children arriving at emergency departments across the UK would normally be given 20 ml per kg of fluid bolus therapy very quickly to help save their lives.

Doctors use 20 ml per kg per fluid bolus because of previous research; however this research did not involve any large scale clinical trials so it is weak evidence. A recent very large African trial called FEAST (Fluid Expansion as Supportive Therapy) involved 3000 children and showed that 20 ml per kg may be too much fluid bolus therapy and may be harmful. FEAST showed that using less fluid bolus therapy might be a better in treating children with septic shock. However, as FEAST was conducted in a poor income country with very limited resources, and other supportive treatments routinely given in richer countries were not available, it is not clear whether the same results would happen in a richer country like the UK. Further research is needed to find out which amount is best at treating children with septic shock in the UK.

3) How was it decided which fluid amount my child/relative received?

FiSh is a randomised controlled trial, which means that each child is randomly put into one of two groups (e.g. children allocated to group 1 receive 20ml per kg of fluid per fluid bolus and children allocated to group 2 receive 10ml per kg of fluid per fluid bolus). To make it fair the groups are selected by a computer programme. Your child/relative had an equal chance of receiving either 10 ml per kg or 20 ml per kg per fluid bolus. Before entering the study your child/relative would have normally received 20 ml per kg of fluid bolus as part of standard treatment. Your child/relative would only have been entered into the study if the team looking after him/her thought fluid bolus therapy was necessary.

Your child's progress has been closely monitored and they have received all other treatments they needed to give the best chance to recover from severe infection.

4) Why am I being asked *after* my child/relative has been given the treatment rather than before?

As this was a medical emergency, we could not delay giving the fluid boluses your child/relative needed. Explaining the study to you in advance would have caused a delay in giving your child/relative urgent treatment. We have therefore come to talk to you about the study as soon as possible after the medical emergency. This is called "research without prior consent". This method of consent has been used in other emergency studies. We are asking for your permission to collect information for the study about your child/relative's hospital stay. This information will help the study team find out which of the two amounts of fluid works best in treating children with septic shock.

5) What will happen next?

If you agree for your child/relative to be part of the study, the FiSh team will collect anonymised information about your child/relative's health and hospital stay, which will be sent to the Intensive Care National Audit & Research Centre (ICNARC) where it will be held securely. The doctors and nurses will continue to monitor your child/relative closely and treat them according to current best practice.

If you do not agree for your child/relative to be part of this study, your child/relative's information will not be used. This will not change the care they will receive. You can change your mind at any time and can contact the research team using the contact details on the first page of this sheet. The study results will be made available on the study website when the study is finished.

6) Who is involved in this study?

The study is being run in 12 Accident and Emergency Departments and 3 Paediatric Intensive Care Units across the country. The National Institute for Health Research (NIHR) Health Technology Assessment (HTA) programme is funding the study. Dr David Inwald (Imperial College Healthcare NHS Trust) is the FiSh Chief Investigator. The FiSh research team are qualified to do this study because they have all the specialties and skills needed. Members of team have a lot of experience in caring for children with septic shock and are very active in health research. Parents of children who have experienced septic shock have been involved in the development of this study, including this information sheet and how you were asked to take part.

7) What if there is a problem?

Complaints: If you have a concern about any aspect of the FiSh Pilot Study, you should ask to speak with the research team (contact details are on the first page) who will do their best to answer your questions. If you remain unhappy and wish to complain formally, you can do this through the NHS Complaints Procedure – details can be obtained from your child/relative's hospital.

Harm: Imperial College Healthcare NHS Trust holds standard NHS Hospital Indemnity and insurance cover with NHS Litigation Authority for NHS Trusts in England, which apply to this study. If you experience serious and enduring harm or injury as a result of taking part in this study, you may be eligible to claim compensation without having to prove that Imperial College Healthcare NHS Trust is at fault. This does not affect your legal rights to seek compensation. If you are harmed due to someone's negligence, then you may have grounds for a legal action. Regardless of this, if you wish to complain, or have any concerns about any aspect of the way you have been treated during the course of this study then you should immediately inform the Investigator. The normal National Health Service complaints mechanisms are also available to you. If you are still not satisfied with the response, you may contact the Imperial AHSC Joint Research Compliance Office.

8) Who has reviewed the study?

The study was given Research Ethics Committee (<*insert REC NUMBER*>) favourable ethical opinion.
