# Supplementary material for: A qualitative feasibility study to inform a randomised controlled trial of fluid bolus therapy in septic shock
Source: Arch Dis Child. 2017 Aug 28;103(1):28–32. doi: 10.1136/archdischild-2016-312515 (PMC5754873; doi:10.1136/archdischild-2016-312515)
Supplement: Supplementary file 3 [file archdischild-2016-312515supp003.docx]

**Table 2.** Approach to qualitative data analysis

| Phase | Description |
| --- | --- |
| Transcription and data cleaning | Interviews transcribed by a professional company (Voicescript) were checked for accuracy. Personal information (*e.g.,* names) were removed to ensure anonymity. |
| Familiarising with data and generating initial themes | CBO read and re-read a sample of transcripts noting down initial ideas around main themes and potential codes. |
| Developing the coding framework | Using NVivo 10 Software, CBO created codes for initial main themes (*e.g.,* thoughts on FiSh; thoughts on FiSh PIS; thoughts on RWPC; decision-making in the emergency setting; and misunderstandings and misconceptions) and began developing a coding framework using line by line coding, comparing between transcripts as part of a constant comparative approach ^29 30 31^. |
| Initial coding meeting | CBO and KW met to discuss early themes and develop the coding framework. |
| Second coding | KW second coded a sample (2 bereaved, 1 non-bereaved; 15%,) of transcripts and made notes on any new themes identified and how the framework could be refined. |
| Second coding meeting | CBO and KW met to discuss, reflect and refine the specifics of each theme in the coding framework^26^. |
| Completion of coding of transcripts | CBO completed coding of all transcripts in preparation for write-up. |
| Write-up and final revision of coding | CBO and KW developed the manuscript using themes to relate back to the study aims ensuring key findings and recommendations were relevant to the FiSh trial design and site staff training (*i.e.,* catalytic validity) ^31 34^. Final discussion and development of selected themes occurred during the write-up phase. |
