# Supplementary material for: A qualitative feasibility study to inform a randomised controlled trial of fluid bolus therapy in septic shock
Source: Arch Dis Child. 2017 Aug 28;103(1):28–32. doi: 10.1136/archdischild-2016-312515 (PMC5754873; doi:10.1136/archdischild-2016-312515)
Supplement: Supplementary file 4 [file archdischild-2016-312515supp004.docx]

**Table 3.** Selected quotations from parents by theme

| Theme | Sub-theme | Example quotes |
| --- | --- | --- |
| RWPC is acceptable but some initial concerns | Logical solution | *At first I was like what? You’d do that without even finding out if it was okay? And then I kind of thought about it and actually that does make sense* (P15, mother, bereaved).  *I had absolutely no problem with it [RWPC]* (P2, mother, non-bereaved, prior experience of RWPC).  *It totally makes sense because as a parent that’s been through it, if they had come and asked us lots of different questions it probably would have brought up a lot more questions, which would have resulted in the subsequent delay in treating [child's name]… and at that time there wasn’t a lot of time to kind of faff about telling us things. So yeah, it totally makes sense* (P15, mother, bereaved). |
|  | Acceptability dependent on nature of the trial | *It depends what they’re actually doing… When it’s your child, I think as long as it isn’t sort of sold as part of an experiment* (P6, mother, non-bereaved).  *I mean I don’t have any issues with it. The only concern would be that I would want to know if you’ve done something without my consent has there been an impact on him in a negative way… Would it have altered anything? And if you can give me assurances around that I can’t see that there's any risk either way* (P10, mother, non-bereaved). |
| Support for FiSh but some concerns and misconceptions | Outcome dependent | *I wouldn’t have had a problem at all if someone had come to me a few hours afterwards and said, “Can we use the information?”, …I’d have been quite happy that she’d have been involved in the trial because I know that obviously it’s worked and she’s better* (P4, mother, non-bereaved).  *Personally, if she had stabilised then yeah, that would be fine… If I thought she was touch and go and she was being used as a trial perhaps then I would feel hang on a minute… Because you want anything you can cling onto. A reason or anything. So I would think what is the reason that she didn’t survive, was it because she was given 10 and not 20?* (P19, mother, bereaved). |
|  | Misconception: current practice is proven to be effective | *I think you just want the tried and tested don’t you? It’s the difference between saying well we can put a plaster on this, or not put a plaster on it. I would be like well, what do you normally do? Well, we normally put a plaster on it. Well, put a plaster on it then* (P6, mother, non-bereaved).  *Obviously we know 20 mL/kg works… That’s the thing isn't it? It’s like well why are you changing it if it already works* (P21, mother, bereaved)? |
|  | Misconception: more fluid is better | *To me it was just give her as much as you can, that’s what was in my mind. They kept saying another bolus, another bolus. In my mind, that was what was saving her life* (P1, mother, non-bereaved).  *Ultimately at a rate of doing it 20mls per kilo, obviously they might stabilise faster* (P21, mother, bereaved)*.* |
|  | Tailored explanation is important to address concerns and misconceptions | *That’s quite important, I think… reiterating that the fluid treatment is part of a package of things and that it’s not necessarily the be all and end all of the treatment* (P17, mother, bereaved).  *I think hearing those two points, the two key points around there being limited or no evidence really that the way we're doing it is the right way… I think that would definitely sway me to take part* (P2, mother, non-bereaved).  *I’d be quite happy that they’d keep an eye on things and keep monitoring closely* (P4, mother, non-bereaved). |
|  | Unclear or missing study information | *I mean it's changed when you said he would have had the standard treatment anyway and then they would go on to the trial. I suppose if it's emphasised that everyone gets the same initial treatment, the emergency treatment… that would be easier* (P16, father, bereaved).  *If he was randomly selected to only have the 10 mL which is, you know, not with what the guidelines go with at the moment, would he have been constantly monitored and would that amount have been increased if they thought it was necessary. Or would they have just rigidly stuck to their guns? That sort of thing I think would have run through my mind (P17, mother, bereaved).*  *Does it say anywhere what the bolus actually does? That might be something, 'cause I was thinking I don't really know. I clocked that the idea is to reduce it but not actually what it does* (P13, mother, non-bereaved).  *I just wondered if the 20 mL has to be given over a certain amount of time?* (P17, mother, bereaved). |
|  | Not voicing concerns | *I think generally… your child's ill, somebody takes them off you and knows what they're doing, you just kind of let them deal with it. You don’t get too involved other than just asking how they're doing, but you don’t necessarily voice concerns* (P5, mother, non-bereaved).  *I don't think it would be at the forefront of what's going on to question the doctor* (P11, mother, non-bereaved)*.* |
| Approaching non-bereaved parents for a RWPC discussion | Timing | *I think it'd be very much dependent on the actual time… if I was asked this once my child was stable, I'd know…he's okay… before that point I'd have probably found it quite hard to make any decisions* (P5, mother, non-bereaved).  *Being in that stressful situation, I don't think it would benefit the parent or the staff that are having to ask these questions… Once a parent's a bit calmer, it literally will open up a whole different world to you guys* (P11, mother, non-bereaved).  *The real first 24 hours might be too soon possibly, because emotions are running high and you know, there's a lot of new, strange things happening. It might be a little bit much to ask at that point* (P7, father, non-bereaved). |
|  | Gauge on a case-by-case basis | *I think that’s probably where it would be down to each individual, you know, case by case… it’s always going to be a difficult position but people react completely different to their child being in that sort of environment… So I think it would be about the timing, and some degree of judgement on behalf of the medical staff to actually ask can this person reasonably give consent at this time* (P10, mother, non-bereaved). |
| Approaching bereaved parents for a RWPC discussion | Prepare for parental concerns about RWPC | *I think probably my only concern is, as a bereaved parent, you are always looking for a reason as to why it happened. And it possibly would lay the door open for people to go you did it wrong, you shouldn’t have done that. That’s my only concern. It gives you angle - it’s the doctor's fault because they did this and they didn’t ask me* (P21, mother, bereaved). |
|  | RWPC is acceptable as a way to help others | *If it helps one other family, it’s fine by us. At the end of the day that’s what it’s about. We can’t bring our [child] back and if this helps one other child not go through the suffering, the pain, that’s fine by us. I would just say just get on with it. Just do it* (P14, father, bereaved).  *To be honest, I am so desperate to help, or if [child's name] information can be used in any way... If it could help stop it happening to someone else, or help understand why, I would be happy for them to use it for research without confirming it first. For me, I can’t think of any negatives* (P19, mother, bereaved). |
|  | Do not approach in hospital | *I don’t think you should do it straight away. I don’t think it should be done in hospital. It’s just an overwhelming experience is all I can say. And then to have that, you won’t take it in, you won’t understand. You'll be more angry that your child is dead and they did this and they didn’t tell you* (P21, mother, bereaved).  *On the day, I think you’re more likely to get parents who will want to opt-out. And I think that you will get more of an opt-out the earlier that you do it* (P17, mother, bereaved). |
|  | Acceptability of postal contact and ‘opt out’ approach | *I think a letter is the right way round but with a lot of sensitivity in the way it's worded because it will come as a shock to families* (P18, father, bereaved).  *I think the opt-out rather than the opt-in is fair because I think if it's an opt-in situation, you're going to lose the data. And I think by opting-out you give the people, if they have strong feelings the other way, you give them that option* (P16, father, bereaved).  *Could you make that in like bold or underlined or something, that they will be included? Because it’s quite often that when you receive something like that you’re like well if you don’t get in touch then that’s it, you don’t get included. Whereas you’re saying actually if we don’t get in touch with you, you are being included in it. And I think, yeah maybe that little bit could be highlighted* (P15, mother, bereaved). |
|  | Importance of parent – practitioner relationships | *Depending on your stay, you become quite familiar with certain nurses and you do have a rapport with them. And I think some, from our point of view, some nurses we would definitely have accepted letters from them probably a bit better than from other nurses* (P15, mother, bereaved)*.* |

FiSh, Fluids in Shock; RWPC, Research without prior consent.
