# Supplementary figures and images for: A qualitative feasibility study to inform a randomised controlled trial of fluid bolus therapy in septic shock
Source: Arch Dis Child. 2017 Aug 28;103(1):28–32. doi: 10.1136/archdischild-2016-312515 (PMC5754873; doi:10.1136/archdischild-2016-312515)

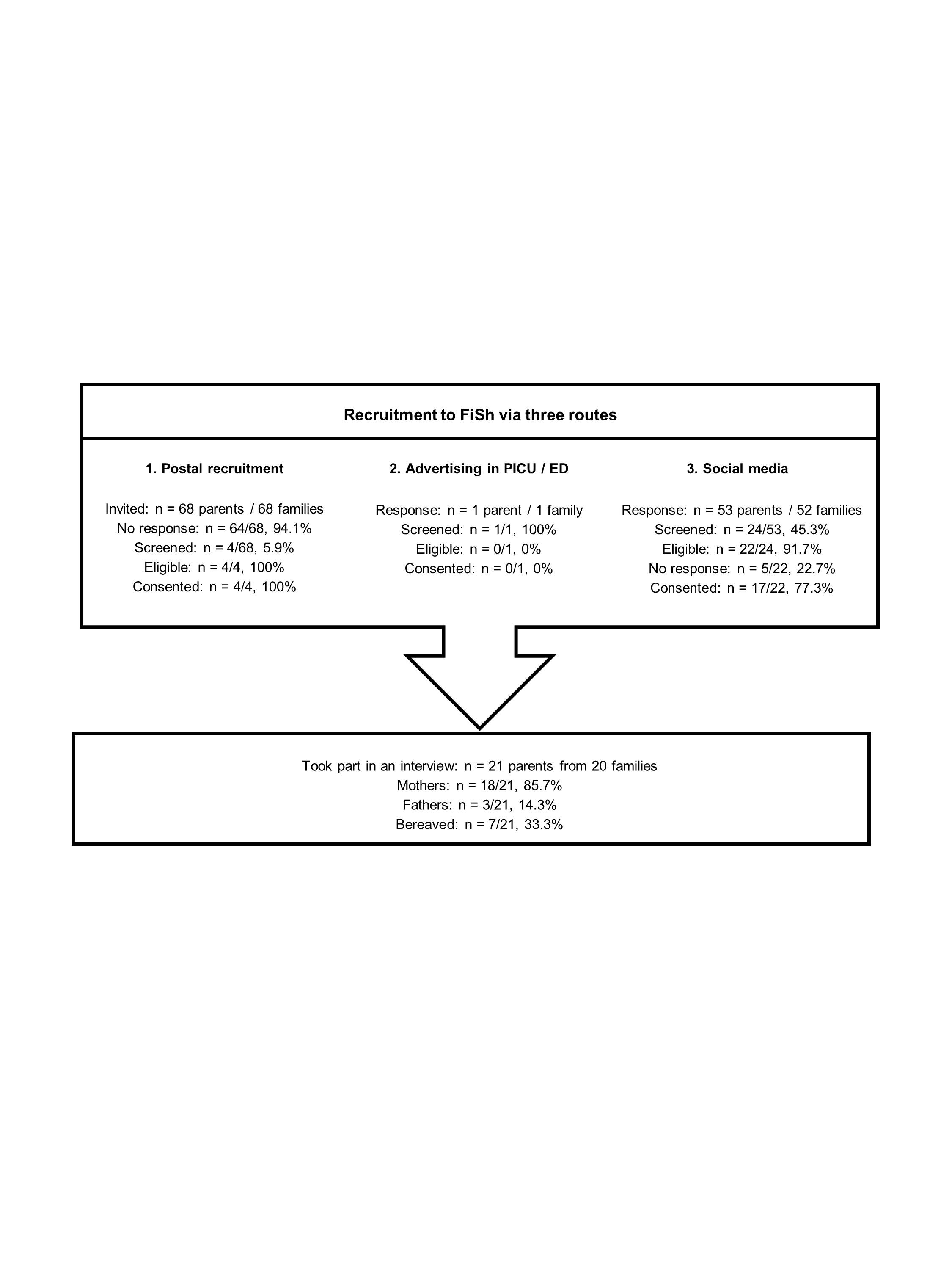

Supplement: Supplementary file 5 [file archdischild-2016-312515supp005.jpg]
